# Altered Weibull Degree Distribution in Resting-State Functional Brain Networks Is Associated With Cognitive Decline in Mild Cognitive Impairment

**DOI:** 10.3389/fnagi.2020.599112

**Published:** 2021-01-05

**Authors:** Yifei Zhang, Xiaodan Chen, Xinyuan Liang, Zhijiang Wang, Teng Xie, Xiao Wang, Yuhu Shi, Weiming Zeng, Huali Wang

**Affiliations:** ^1^College of Information Engineering, Shanghai Maritime University, Shanghai, China; ^2^State Key Laboratory of Cognitive Neuroscience and Learning and IDG/McGovern Institute for Brain Research, Beijing Normal University, Beijing, China; ^3^Beijing Key Laboratory of Brain Imaging and Connectomics, Beijing Normal University, Beijing, China; ^4^Dementia Care and Research Center, Peking University Institute of Mental Health (Sixth Hospital), Beijing, China; ^5^Beijing Key Laboratory for Translational Research on Diagnosis and Treatment of Dementia, Beijing, China; ^6^National Clinical Research Center for Mental Disorders (Peking University Sixth Hospital), Beijing, China

**Keywords:** Alzheimer's disease, mild cognitive impairment, resting-state functional MRI, degree distribution, Weibull, network

## Abstract

The topological organization of human brain networks can be mathematically characterized by the connectivity degree distribution of network nodes. However, there is no clear consensus on whether the topological structure of brain networks follows a power law or other probability distributions, and whether it is altered in Alzheimer's disease (AD). Here we employed resting-state functional MRI and graph theory approaches to investigate the fitting of degree distributions of the whole-brain functional networks and seven subnetworks in healthy subjects and individuals with amnestic mild cognitive impairment (aMCI), i.e., the prodromal stage of AD, and whether they are altered and correlated with cognitive performance in patients. Forty-one elderly cognitively healthy controls and 30 aMCI subjects were included. We constructed functional connectivity matrices among brain voxels and examined nodal degree distributions that were fitted by maximum likelihood estimation. In the whole-brain networks and all functional subnetworks, the connectivity degree distributions were fitted better by the Weibull distribution [f(x)~x^(β−1)^e^(−λ*x*^β^)^] than power law or power law with exponential cutoff. Compared with the healthy control group, the aMCI group showed lower Weibull β parameters (shape factor) in both the whole-brain networks and all seven subnetworks (false-discovery rate-corrected, *p* < 0.05). These decreases of the Weibull β parameters in the whole-brain networks and all subnetworks except for ventral attention were associated with reduced cognitive performance in individuals with aMCI. Thus, we provided a short-tailed model to capture intrinsic connectivity structure of the human brain functional networks in health and disease.

## Introduction

Resting-state functional magnetic resonance imaging (rsfMRI) studies have suggested that the human brain can be considered an efficiently integrated network that is divided into several functionally linked subnetworks. Examining the topology of brain networks can provide valuable information about the organization of the networks such as hub regions, robustness levels, and ability to communicate information (Bullmore and Sporns, 2012; Dai et al., 2015; Liao et al., 2017). One of the most important properties describing the network topology is the degree distribution of network nodes, a graph theory property that characterizes the probability distribution of the number of connections between pairs of nodes in a network. In resting-state brain networks, there is no agreement on which model can better describe the degree distribution. One view is that the degree distribution follows the heavy-tailed power law (Van Den Heuvel et al., 2008; Hanson et al., 2016; Forlim et al., 2019) based on the simple growth mechanisms, such as preferential attachment (Barabási and Albert, 1999). Another view is that the degree distribution can be better fitted by a short-tailed distribution such as power law with exponential cutoff (Bassett et al., 2006; Hayasaka and Laurienti, 2010; Cao et al., 2016) and Weibull distribution (Nakamura et al., 2009; Gupta and Rajapakse, 2018), considering the wiring-cost constrains (Bullmore and Sporns, 2012) in the human brain. These suggest that power law, power law with exponential cutoff (also called truncated power law), and Weibull distribution (also called stretched exponential) are the three most frequently reported models for fitting the degree distribution of human brain networks, but the findings are not conclusive.

Amnestic mild cognitive impairment (aMCI) is an intermediate stage between healthy aging and (most likely to develop into) Alzheimer's disease (AD) (Petersen et al., 2001). For elderly subjects with cognitive impairment, rsfMRI is acquired without engaging the subjects in a particular cognitive task (i.e., during rest) and therefore has the advantages of clinical practice. It has been argued that neuropsychiatric disorders including AD can be considered as “dysconnectivity syndrome” and that a combination of graph theory method enables a quantitative study of the topology of the network (Bullmore and Sporns, 2009; Xie and He, 2012). In resting-state networks, functional connectivity (FC), the synchronization of spontaneous low-frequency fluctuations in brain activity between different brain regions, is the most commonly used measure of the number of connections in a degree distribution. Alterations of FC in resting-state networks have been identified in the early stages of AD, including elderly cognitively normal subjects with increased amyloid-beta (Aβ) level and aMCI patients (Hedden et al., 2009; Wang et al., 2013a; Zhang et al., 2016). Such aberrant FCs were observed even when controlling for gray matter atrophy (Sorg et al., 2007; Agosta et al., 2012; Wang et al., 2013b). Together, these results suggest that alterations of degree distribution in resting-state networks could be occurred in AD.

In the prodromal stage of AD, changes in network structures are usually accompanied by a variety of alterations in cognitive functions, such as memory, attention, and executive functions. Previous rsfMRI studies suggest that brain regions involving the medial and lateral prefrontal and parietal cortices, insula, and thalamus are preferentially affected in AD (Buckner et al., 2009; Dai et al., 2015, 2019). Two recent meta-analyses have explored resting-state brain changes in the progression of AD and overlaid the coordinates of these changes onto functional subnetworks. In the meta-analysis of Li et al. (2015a), they included 25 resting-state and 75 task-based fMRI studies, and the results revealed that compared to healthy controls, MCI patients showed altered brain activities in default, frontoparietal, and limbic networks during rest; when fulfilling cognitive tasks, there were also abnormalities in ventral attention and somatomotor networks in addition to these three networks. Including 40 resting-state fMRI studies, Badhwar et al. (2017) found that MCI and AD patients showed connectivity alterations in default, salience, and limbic networks. However, very few studies have examined AD-related changes in the topology architecture of functional subnetworks as described by the degree distribution.

Areas commonly activated during complex cognitive tasks are distributed across several of the classic resting-state networks (Li et al., 2015b). For example, a meta-analysis showed widespread memory-related activities across temporal, frontal parietal, and other regions of the brain (Spaniol et al., 2009). Also, these areas can also be observed to have functional synchronization during rest (Zhang et al., 2016). Therefore, to understand changes in degree distributions and whether they are related to cognitive performance, not only the connections within a specific functional subnetwork need to be considered, but also the connections of that network to other regions in the whole brain. Many studies have also found that resting-state FC changes, both within and in between the classic networks, are significantly associated with the patients' cognitive performance (Dai et al., 2015; Pasquini et al., 2015; Zhang et al., 2016). However, most of these studies have focused on some specific functional connections. The changes in the degree distributions of global connections in different functional subnetworks remain to be elucidated.

To address these issues, we used rsfMRI to investigate connectivity degree distributions of nodes in the whole-brain functional networks and within functional subnetworks in aMCI and healthy controls. The seven referenced functional subnetworks were defined according to Thomas Yeo et al. (2011). Using the three most likely candidate models (power law, power law with exponential cutoff, and Weibull distribution), we sought to determine whether degree distributions of these networks (1) can be better fitted by one of the candidate models, (2) are altered in aMCI, and (3) are associated with cognitive performance as assessed by standard neuropsychological tests.

## Materials and Methods

### Participants

Seventy-one subjects (30 aMCI patients and 41 healthy controls, HC) were included in this study. The aMCI patients were recruited from the Dementia Care and Research Center (memory clinic) at Peking University Sixth Hospital. Elderly cognitively HC were screened from local communities. All participants' demographic information was collected through detailed clinical consultations, including age, sex, education level (years of education), history of depression, treatment information, and current medication use. All participants were right-handed, drug-naive, and aged between 55 and 85 years. All participants received a neuropsychiatric and neuropsychological examination, and a geriatric psychiatrist provided a definitive diagnosis and Clinical Dementia Rating (CDR) (Chan and Siu, 2005) score after a clinical interview. Participants with aMCI met Petersen's MCI criteria (Petersen, 2004), which are as follows: (1) complaints of memory problems that are confirmed by an informant, (2) preserved general cognitive function (Mini-Mental State Examination (MMSE) (Folstein et al., 1975) scores ≥ 24), (3) intact or mildly impaired daily living ability (an ADL score ≤ 26) (Lawton and Brody, 1969), and (4) do not meet the diagnosis of dementia (World Health Organization, 2010). All HC had no cognitive complaints and did not meet clinical criteria for cognitive impairment or depression. MMSE cutoff scores were ≥24 for the HC group. The requirement for global CDR was ≤ 0.5 in the aMCI group and 0 in the HC group. For all participants, exclusion criteria were as follows: (1) history of stroke, tumor, subdural hematoma, other cerebrovascular disease or intracranial space-occupying disease, and obvious risk factors for cerebrovascular disease, (2) currently taking anti-dementia or antidepressant medications, (3) history of drug or substance abuse, (4) history of neurological or psychiatric disorders, and (5) presence of a physical illness that may affect cognition or emotion. Written informed consent was obtained from each participant, and this study was approved by the Medical Research Ethics Committee of Peking University Sixth Hospital, Beijing, China. The data of eight subjects were discarded during scanning, preprocessing, diagnosis, or analysis (for details, see Supplementary Figure 1). Neuropsychological and demographic summary statistics for each diagnostic group are provided in Table 1. No significant between-group differences were found in gender and education level (gender: *p* = 0.45; education level: *p* = 0.81). The age of the HC group was significantly lower than that of the aMCI group (*p* < 0.01). The MMSE and the Montreal Cognitive Assessment (MoCA) (Nasreddine et al., 2005) scores in the HC group were significantly higher than those in the aMCI group (ps < 0.001).

**Table 1:** Characteristics of each diagnostic group.

**Variable**	**HC**	**aMCI**	***p*-value**
N	41	30	-
Gender (F/M)	28/13	17/13	0.45^b^
Age (y)	70.3 [7.0]	74.8 [5.7]	<0.01^a^
Education	4.3 [0.9]	4.2 [1.1]	0.81^a^
MMSE	28.5 [1.3]	26.7[1.9]	<0.001^a^
MoCA	25.7 [2.8]	22.2 [3.5]	<0.001^a^

*Values represent the mean [standard deviation] or number of subjects. HC, cognitively healthy; aMCI, amnestic mild cognitive impairment; N, number of subjects; F, female; M, male; MMSE, Mini-Mental State Examination; MoCA, Montreal Cognitive Assessment*.

a *The P-value was obtained by the two-sample two-tailed t-test*.

b *The P-value was obtained by the two-tailed Pearson χ^2^ test*.

### Assessment of Cognitive Ability

Cognitive ability was quantified by a mean score of the MMSE and the MoCA. The MMSE and the MoCA are two widely used screening assessments for detecting cognitive impairment. Both tests have a 30-point questionnaire and cover a wide range of cognitive functions, with the MMSE testing dysfunctions of attention and calculation, recall, language, orientation, abilities to repeat named prompts and to follow simple commands, and the MoCA screening for dysfunctions of attention, executive function, language, visual, memory, abstracting thinking, structure calculation, and directional force.

### MRI Acquisition

All MRI data were acquired on a 3T Siemens Magnetom Prisma scanner (Siemens, Erlangen, Germany). Foam pads and headphones were used to minimize head movement and scanner noise. For rsfMRI, the following acquisition parameters were used: T2^*^-weighted multi-band echo planer imaging (EPI) pulse sequence in transverse slice orientation, with multiband acceleration factor of 4, repetition time (TR)/echo time (TE) = 500/30 ms, flip angle (FA) = 47°, field of view (FOV) = 231 × 231 mm^2^, matrix = 66 × 66, slices = 36, thickness = 3.5 mm, voxel size = 3.5 × 3.5 × 3.5 mm^3^, echo spacing = 0.4 ms, and bandwidth = 3,444 Hz/pixel. The subjects were instructed to keep their eyes closed but not fall asleep, relax their minds, and minimize their movement during data acquisition. rsfMRI scan lasted for 480 s and included 960 functional volumes for each subject. T1-weighted magnetization-prepared rapid gradient echo (MPRAGE) sagittal images were also scanned with the following sequence: TR/TE = 2,530 ms/2.98 ms, FA = 7°, inversion time = 1,100 ms, FOV = 256 × 224 mm^2^, slices = 192, thickness = 1 mm, voxel size = 0.5 × 0.5 × 1 mm^3^.

### Image Pre-processing

The first 10 rsfMRI volumes were discarded to ensure steady-state magnetization. The remaining volumes were then realigned to the first volume to correct for head motion. Subjects were excluded if the head motion is larger than 3 mm and 3°. The mean functional image after motion correction was coregistered to the individual T1-weighted images using a linear transformation (Collignon et al., 1995) and were then segmented into gray matter (GM), white matter (WM), and cerebrospinal fluid (CSF) with a priori tissue maps of SPM by using a unified segmentation algorithm (Ashburner and Friston, 2005). The resultant GM, WM, and CSF images were further non-linearly registered into the Montreal Neurological Institute (MNI) space with the information estimated in unified segmentation and then averaged across all subjects to create custom GM, WM, and CSF templates. Then, the custom templates were used as reference images to segment the coregistered T1 images for the second time. This two-step registration procedure based on custom template could minimize the inaccuracies of the spatial normalization of rsfMRI volumes caused by GM atrophy in elderly people. The transformation parameters estimated during unified segmentation were applied to motion-corrected rsfMRI images and then the images resampled to 3-mm isotropic voxels, which reflect the neuronal pattern of the columnar grain (Kriegeskorte et al., 2010) and are the minimum spatial resolution to capture cortical folding (Kiselev et al., 2003). Subsequently, a linear trend was removed and 24 head motion parameters, mean global signal, and the WM and CSF time courses were regressed out from the spatially normalized rsfMRI scans. A bandpass filter was used to remove frequencies outside of the 0.01–0.1-Hz range. It should be noted that no spatial smoothing was applied to the rsfMRI time series, avoiding local artificial correlations between voxels. The MATLAB-based Statistical Parametric Mapping (SPM12, Wellcome Department of Cognitive Neurology, London, http://www.fil.ion.ucl.ac.uk/spm/) and graph theoretical network analysis toolbox (GRETNA, Beijing Normal University, http://www.nitrc.org/projects/gretna/) (Wang et al., 2015) were used to carry out all functional imaging data pre-processing.

### Degree Distribution Analysis

#### Creating Brain Networks and Degree Calculation

The degree distribution analyses were based on binarized brain networks according to the original definition of nodal degree (i.e., the number of binary edges of a node). To establish the whole-brain networks, for each subject, FC matrices were computed by Pearson's correlation between the times series of any pairs of brain voxels. This procedure was constrained within a GM mask (N_voxels_ = 47,294) generated by thresholding (cutoff = 0.2) the mean GM probability map of all subjects. A threshold T between 0.4 and 0.6 (with steps of 0.1) defined links between any pairs of nodes (voxels) in the networks. The maximum T value was empirically set to 0.6 to maintain the network integrity, minimizing the number of disconnected voxels, and the minimum value was set to 0.4 to keep the small-world property of the networks and to ensure that the matrices were sufficiently sparse for voxel-based networks (Van Den Heuvel et al., 2008). For each given voxel, *i*, its degree was calculated by the following equation:

$$\begin{array}{l} degree\left(i\right)=\overset{N_{voxels}}{\underset{j=1,\;j\neq i}{\sum }}a_{ij},\;\{\begin{array}{c} a_{ij}=1\;if\;r_{ij}\geq T\\ a_{ij}=0\;if\;r_{ij}<T \end{array}\;, \end{array}$$

where *r*_*ij*_ was the correlation coefficient between voxel *i* and voxel *j*. The seven referenced functional subnetworks were obtained from previous studies based on the rsfMRI data from 1,000 participants and a data-driven clustering approach (Thomas Yeo et al., 2011) (see Supplementary Figure 2), including visual (V), sensorimotor (SM), dorsal attention (DA), ventral attention (VA), limbic (Lim), frontoparietal (FP), and default mode (DM). For each subnetwork, nodal degrees were still computed by summing the connections of a voxel to any other voxels in the whole brain (not only the connections within the subnetwork), which we refer to here as the global degree of nodes in a particular subnetwork.

For validity reasons, to identify the whole strength pattern of degree distribution in both the HC and aMCI groups, we performed a functional connectivity strength (FCS) analysis, also called degree centrality of a weighted network (Buckner et al., 2009; Zuo et al., 2012; Dai et al., 2015). For each subject, we built whole-brain FC matrices by computing Pearson's correlations between the time series of any pairs of brain voxels. This process was constrained within the same GM mask. For each voxel, *i*, the FCS was computed by the following equation:

$$\begin{array}{l} FCS\left(i\right)=\frac{1}{N_{voxels}}\overset{N_{voxels}}{\underset{j=1,\;j\neq i}{\sum }}z_{ij},\;r_{ij}>r_{0}\;, \end{array}$$

where *z*_*ij*_ is the Fisher's z-transformation of *r*_*ij*_, *r*_0_ is a threshold that eliminates weak correlations possibly arising from noise [here *r*_0_ = 0.2, based on a previous study that evaluated different thresholds (Dai et al., 2015)].

Notably, only positive correlations between voxels were considered in the calculation of the nodal degree and FCS; connectivity terminating within 20 mm of each source voxel center was set to zero to avoid potential shared signals between nearby voxels. These voxel-wise brain network analyses were performed using an in-house toolbox (developed by Dr. Mingrui Xia, Beijing Normal University).

#### Degree Distribution Fit

Based on published literatures, we chose three most likely models, including power law, power law with exponential cutoff, and Weibull, as candidate models for the fittings of the degree distributions (see Table 2 for their probability density function). The fittings of the alternative models and the estimations of the model parameters followed the statistical methods from previous study (Clauset et al., 2009) and used the *powerlaw* Python package (Alstott and Bullmore, 2014) (https://github.com/jeffalstott/powerlaw). In general, the visual form of the Complementary Cumulative Distribution Function (CCDF) is more frequently preferred than that of the Probability Distribution Function (PDF) against fluctuations due to finite sample sizes (Clauset et al., 2009). In the fitting procedure, for each network, a vector containing voxels' degrees was sorted in ascending order for each correlation threshold. For every generated network, the maximum likelihood estimation method was used to estimate model parameters. The obtained degrees could only take values in integers. For the power law distribution, there is no exact closed-form expression for the maximum likelihood estimator of the parameter α in the discrete case. The *powerlaw* Python package uses an analytic estimation of α with the method mentioned by Clauset et al. (2009) that provides a faster way to obtain a more precise estimation. The approximate expression of α is

$$\begin{array}{l} \hat{\alpha }≃1+n{\left[\overset{n}{\underset{i=1}{\sum }}ln\frac{x_{i}}{x_{min}-\frac{1}{2}}\right]}^{-\;1}, \end{array}$$

where *x*_*i*_, *i* = 1⋯*n* are the observed voxels' degree values in the vector. In practice, the power law tends to apply only when the values of empirical phenomena are greater than some minimum value *x*_min_. Thus, when initially fitting with the power law, the optimal value of *x*_min_ was obtained by selecting the one that resulted in the minimal Kolmogorov–Smirnov distance between the data and the fit. The initial fit showed that the CCDF of degree distributions of functional brain networks was curved on the double logarithmic axis, and the selection of different optimal *x*_min_ values resulted in a large shift of the fitted lines on the sample curves for different subjects. Thus, the power-law model might not be appropriate. The *x*_min_ was then fixed to 1 in the fittings and comparisons of the other alternative models. Discrete forms of the other alternative models are not defined analytically. Discrete forms of probability distributions are often more difficult to calculate. The *powerlaw* package performs discretization by rounding, summing the continuous distribution to the nearest integer, to calculate approximations to the discrete form of the alternative distribution. For comparison between models, a normalized loglikelihood ratio R and an associated significance value p were used to evaluate the goodness of fit between two competing distributions to identify a better fit. The positive or negative sign of the R indicates which model is better, or a ratio close to zero indicates the two models have similar effects, if the *p*-value is small enough (<0.05). Therefore, we used the mean of *R*-values to determine which model and to what extent is more appropriate at the group level.

**Table 2:** The three candidate models for the fit of the degree distribution.

**Distribution name**	**Probability density function**
Power law	*x*^−α^
Power law with exponential cutoff	*x*^−α^*e*^−λ*x*^
Weibull	*x*^β−1^*e*^−λ*x*^β^^

#### Statistical Analyses

To investigate whether the degree distribution parameter(s) of the best-fit model changed in the aMCI group compared to the HC group, we applied analyses of the following general linear model including diagnosis (HC vs. aMCI), age, gender, and education as independent variables and model parameter as dependent variable:

$$\begin{array}{l} Model\;parameter\;\sim \;\beta_{0}+\;\beta_{1}\times Diagnostic\;group+\beta_{2}\times Age\\ +\beta_{3}\times Gender+\beta_{4}\times Education. \end{array}$$

Then, to detect the relationship between a parameter of a degree model and cognition ability, the parameters of degree distribution that were found significantly altered in the aMCI group were tested as predictors of cognition scores in the aMCI group. We used the following linear regression model, including cognitive ability as dependent variable and model parameter, age, gender and education as independent variables:

$$\begin{array}{l} Cognitive\;ability\;\sim \;\beta_{0}+\;\beta_{1}\times Model\;parameter+\beta_{2}\times Age\\ +\beta_{3}\times Gender+\beta_{4}\times Education. \end{array}$$

For validity reasons, we generated mean FCS maps for the HC and the aMCI groups. The group difference of the FCS maps was evaluated via two-sample *t*-tests controlling for age, gender, and education. A false discovery rate (FDR) procedure was used to correct for multiple comparisons within the GM.

Without other statements, the analyses were corrected for multiple comparisons, FDR-corrected at p = 0.05. All group-level statistical analyses were done with the stats package of statistical software R implemented in R Studio v. 0.98.953 (Boston, MA, https://www.rstudio.com/).

## Results

### Weibull Distribution Fits Brain Networks Better

**Figure 1:**
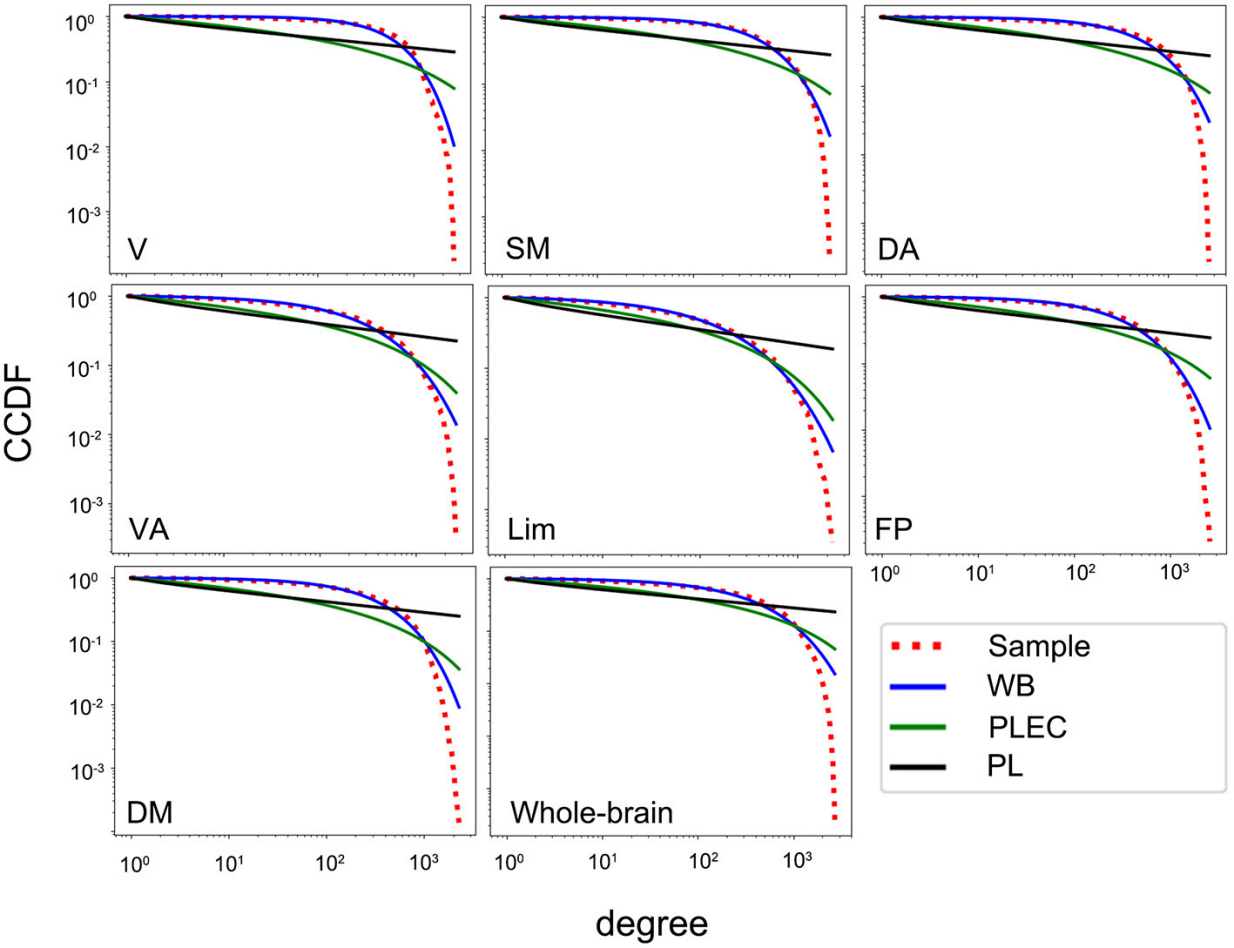
The CCDF plots of the degree distributions for an aMCI subject in the whole-brain network and seven subnetworks and their fit with the three candidate models (correlation threshold *T* = 0.4). V, visual; SM, somatomotor; DA, dorsal attention; VA, ventral attention; Lim, limbic; FP, frontoparietal; DM, default model; WB, Weibull; PL, power law, PLEC, power law with exponential cutoff; CCDF, complementary cumulative distribution function.

In order to estimate connectivity degree distributions of the whole-brain networks and different functional subnetworks, we tested the three candidate models for each subject by using normalized loglikelihood ratios *R* and *p*-values (calculated by R and its standard deviation σ, indicating whether the observed sign of R is statistically significant). A positive R suggests a better fit of the data to the first model, while a negative R suggests a better fit to the second, if the *p*-value is small (< 0.05 here). The results of the group-averaged normalized loglikelihood ratios (only counted if ps < 0.05) for the fittings of degree distributions between two of the three models in the whole-brain networks and subnetworks are listed in Table 3. In the whole-brain networks and all subnetworks, the connectivity degree distributions were fitted better by a Weibull distribution than power law or power law with exponential cutoff. Examples of the CCDF plots and their fittings of the three candidate models in the whole-brain network and the seven subnetworks (correlation threshold T = 0.4) for an aMCI subject are shown (fittings were similar for other subjects and thresholds); see Figure 1. For the fittings compared between Weibull and power law, in all networks, both the HC and the aMCI groups, all of the averaged R ratios were positive and sufficiently larger than zero, indicating that the Weibull is better than the power law in human brain rsfMRI networks. For the 48 times fitting compared between Weibull and power law with exponential cutoff, in all generated networks, 85.4% of the R-values were positive, suggesting that the Weibull is better than the power law with exponential cutoff. While the other 14.6% comparisons have negative R-values, these values were very close to zero, suggesting that the two models fit similarly for these 14.6% comparisons (Table 3). It should be noted that, in general, all R-values tended to decrease as the correlation threshold increases. Consequently, out of the three correlation thresholds, T = 0.4 and 0.5 were more compatible with the Weibull distribution. The reason could be that lower threshold preserves more weak connections. No significant between-group difference in R-values was found in any of the networks.

**Table 3:** Group-averaged loglikelihood ratios between candidate models for the fittings of connectivity degree distributions.

	**WB vs. PL in HC**	**WB vs. PL in aMCI**	**WB vs. PLEC in HC**	**WB vs. PLEC in aMCI**
V	66.9/37.9/25.9	63.8/32.5/21.2	33.8/12.5/7.4	29.8/7.2/2.7
SM	57.6/28.7/18.4	54.8/ 25.0/16.4	27.0/4.2/2.5	23.3/−1.3/0.8
DA	52.6/29.1/18.8	51.1/ 25.9/16.2	25.4/7.5/4.4	23.3/3.8/1.7
VA	48.8/24.9/15.8	46.6/ 22.7/14.8	22.1/4.6/1.6	19.5/0.5/0.5
Lim	43.6/19.0/11.6	40.2/ 16.3/9.8	19.2/−0.1/−1.8	16.5/2.9/2.0
FP	64.8/36.7/22.0	60.7/ 32.2/19.8	34.3/12.3/4.9	29.6/6.8/2.7
DM	80.6/44.5/27.3	75.5/ 39.3/24.6	42.3/13.7/4.8	36.4/7.8/2.2
Whole brain	164.9/87.1/57.2	157.5/ 78.0/51.3	67.3/9.7/4.3	59.0/−2.1/−1.4

*Values represent the mean normalized loglikelihood ratios (ps < 0.05) between candidate models for the fittings of networks generated at threshold of 0.4/0.5/0.6 in the HC and the aMCI groups. V, visual; SM, somatomotor; DA, dorsal attention; VA, ventral attention; Lim, limbic; FP, frontoparietal; DM, default model; WB, Weibull; PL, power law, PLEC, power law with exponential cutoff; HC, cognitively healthy; aMCI, amnestic mild cognitive impairment*.

### Degree Distribution Changes in aMCI

**Figure 2:**
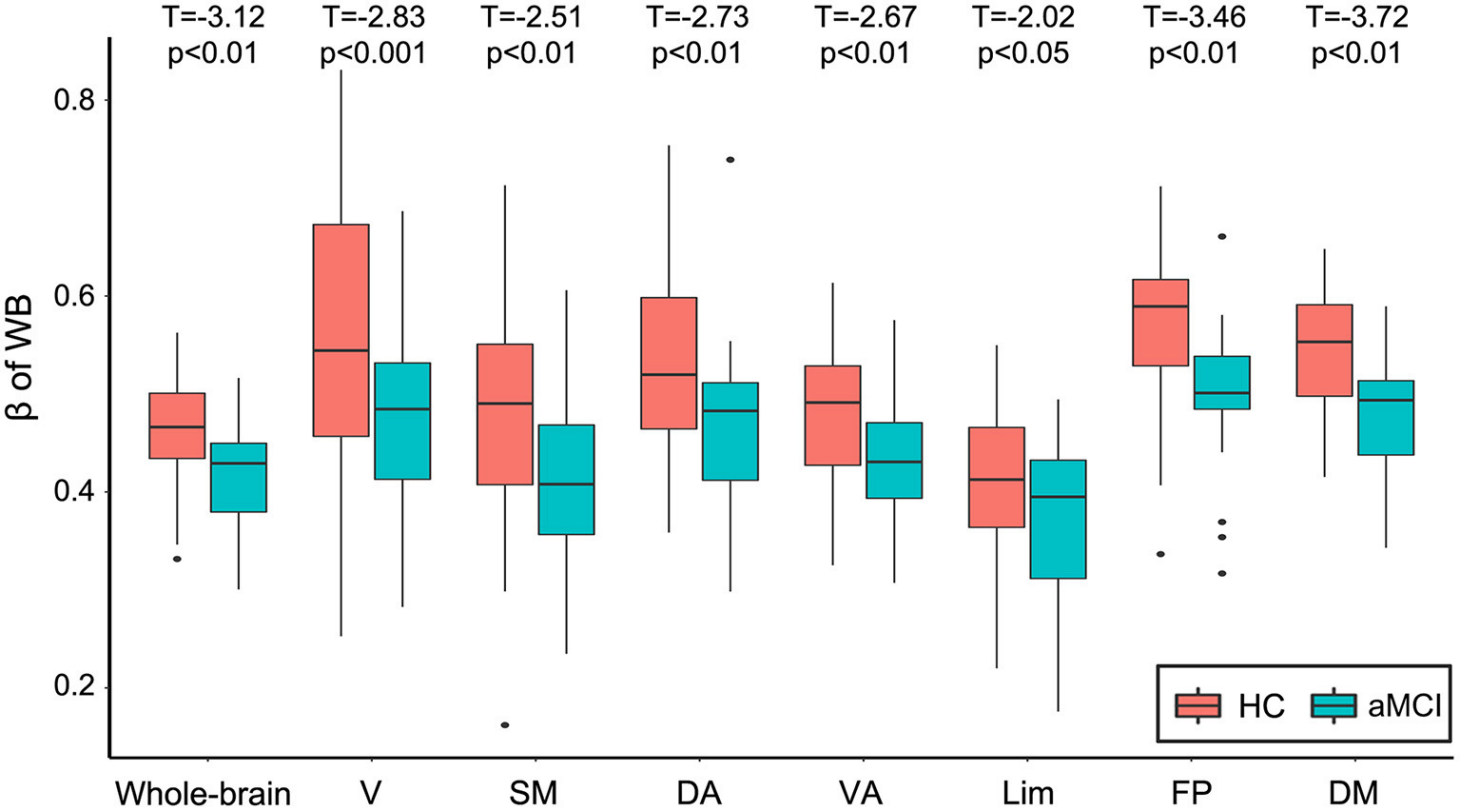
Boxplot of the Weibull β parameters as a function of diagnosis on the degree distributions of the whole-brain network and seven subnetworks (correlation threshold *T* = 0.5). *P*-values of between-group differences were FDR adjusted. V, visual; SM, somatomotor; DA, dorsal attention; VA, ventral attention; Lim, limbic; FP, frontoparietal; DM, default model; HC, cognitively healthy; aMCI, amnestic mild cognitive impairment; FDR, false-discovery rate.

Focusing on the Weibull distribution, we tested whether its two parameters, β and γ, were altered in the aMCI group. The Weibull distribution can be used to describe a distribution between the power law and the exponential function, where the parameter β ∈ (0, 1) denoting the extent it falls between the two distributions. When β = 0, it reduces to a power law distribution and when β = 1, it becomes an exponential distribution. Compared with the HC group, the aMCI group showed lower Weibull β parameters (shape factor) in both the whole-brain network and all the seven subnetworks. When T = 0.4, decreased Weibull β parameters were found in aMCI for connectivity degree distributions in the whole-brain networks (*p* = 0.05) and within functional subnetworks: in FP & DM (ps < 0.01), in V & SM (ps = 0.05), and a tendency in VA (*p* = 0.06, uncorrected *p* = 0.04). When T = 0.5, the β parameters of the Weibull distribution in the whole-brain network and within all seven subnetworks decreased significantly: in V (p = 0.001), in SM, DA, VA, FP, DM, and whole brain (ps ≤ 0.01), and in Lim (*p* = 0.05). When *T* = 0.6, the β parameters were also decreased in V, DA, and the whole brain (ps < 0.05), and a tendency to decrease in SM and DM (ps < 0.1, uncorrected ps < 0.05). All *p*-values reported were FDR-corrected, unless otherwise noted. As shown in Figure 2, the value of β parameters in aMCI were lower than that for HC in the whole-brain network and within all seven subnetworks (correlation threshold T = 0.5). No changes of λ parameter in aMCI were observed compared to HC. For validity reasons, we also examined the group difference of the FCS map between the HC and the aMCI groups (see Supplementary Figure 3). Visual inspection indicated that the spatial distributions of FCS in the aMCI group were similar but weaker than those of the HC group; the FCS of some voxels distributed in regions including the angular and precuneus were increased in the aMCI group. No significant between-group difference was found after an FDR multiple comparisons at p < 0.05 (for the number of connections between voxels in GM).

### Association Between Weibull β Parameter and Cognitive Decline in aMCI

**Figure 3:**
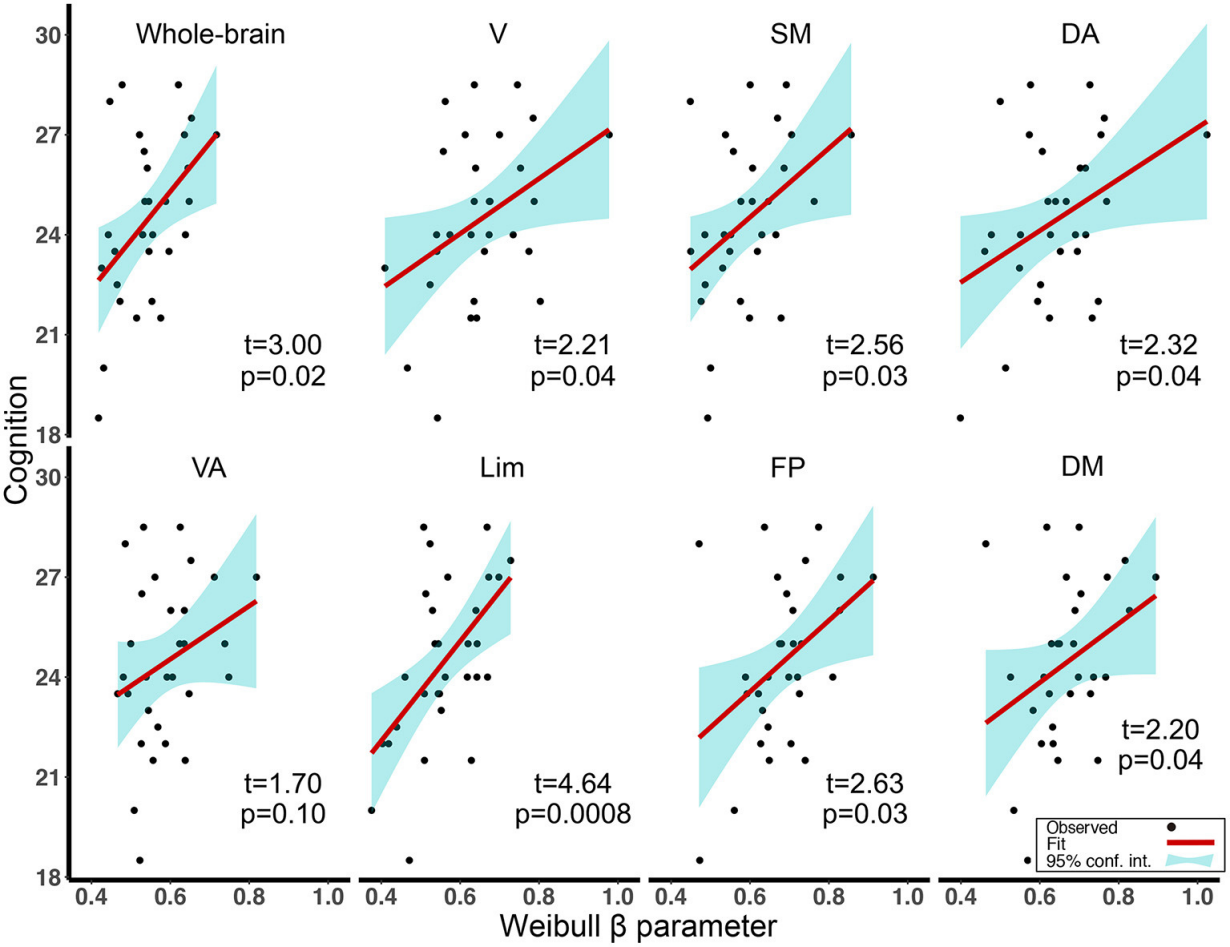
Regression plots of the association between Weibull β parameters and cognitive ability scores for aMCI in the whole-brain network and seven subnetworks (correlation threshold *T* = 0.4). All *p*-values were FDR adjusted. V, visual; SM, somatomotor; DA, dorsal attention; VA, ventral attention; Lim, limbic; FP, frontoparietal; DM, default model; aMCI, amnestic mild cognitive impairment; FDR, false-discovery rate.

Focusing on the Weibull β parameter for which the value was found to be decreased in aMCI, we found lower values of the Weibull β parameter to be associated with reduced cognitive ability in aMCI (mainly in the networks with T = 0.4). Specifically, for connectivity degree distributions in rsfMRI, we found the above associations for the whole-brain network and all subnetworks excluding VA (for the *t-*values and FDR-corrected *p*-values, see Figure 3). We also found the association in Lim with T = 0.5 (t = 2.99, FDR-corrected *p* = 0.04). We computed the diagnosis (HC, aMCI) × Weibull β parameter interactions, controlled for age, gender, and education to correlate cognitive ability in the networks showed the above relationship. The results showed that the slopes of the Weibull β parameter in HC differed significantly from those in aMCI (Supplementary Figure 4).

## Discussion

The first major finding of the present study was that the Weibull distribution fits brain networks better in resting-state fMRI. The second major finding was the decreased Weibull β parameters in the whole-brain network and all seven subnetworks in aMCI subjects compared to HC. The third major finding was that the abnormal decrease in the values of Weibull β parameter in the whole-brain network and the functional subnetworks were associated with reduced cognitive performance in aMCI.

The current finding of Weibull distribution fits brain networks better is in line with previous reports of the nodal degree of human brain functional networks that follow short-tailed distribution such as the Weibull distribution and the power law with exponential cutoff (Nakamura et al., 2009; Hayasaka and Laurienti, 2010; Gupta and Rajapakse, 2018; Zucca et al., 2019). In contrast, the heavy-tailed power law distribution (also called scale-free network) has been extensively discussed (Eguíluz et al., 2005; Van Den Heuvel et al., 2008; Ciuciu et al., 2014; Hanson et al., 2016; Forlim et al., 2019). Most of these studies on the power law had a strong hypothesis that brain networks are structured with simple growth mechanisms, such as preferential attachment (Barabási and Albert, 1999). Under this assumption, the network has scale-free property that allows for efficient communication of information through a few hub nodes. To characterize this property, more complicated network construction methods were adopted in these studies. For example, Hanson et al. (2016) reported brain networks that fit for power law distribution by using a conditional probability-based Bayes network search model that “allows for the node structure to express more subtle hub and modular configurations.” Another rsfMRI study also found that a machine learning-based k-nearest neighbor graph construction of brain networks presents scale-free properties (Forlim et al., 2019). However, a recent study analyzing over a thousand power law distributions from various disciplines concluded that scale-free networks are rare in real-world data, and alternative models such as log-normal often fit degree distributions better than the power law (Broido and Clauset, 2019).

For the present study, we found that the Weibull distribution outperforms the other two commonly reported brain network models in the whole-brain networks and all the seven subnetworks, in both the HC and the aMCI subjects. We agree with the assumption of wiring-cost constrains in human brain (Bullmore and Sporns, 2012). In addition to the fact that the human brain has mechanisms to reduce information processing cost and maximize efficiency, the organization of functional networks is also limited by the spatial structure of the brain. The dynamic properties of functional networks such as their topology and synchronization ability are strongly influenced by small world and other structural connectivity constraints (Bullmore and Sporns, 2009). The architectural constraints prevent the occurrence of long-distance hubs, as the corresponding remote anatomical connections consume more energy. Therefore, on a CCDF plot of the nodal degree with logarithmic axes, the tail of the Weibull distribution may show a downward bend compared to the power law distribution. Notably, it has been suggested that the estimation of the degree distribution is still dependent on several factors such as the pre-processing process, region-based or voxel-based node scale, edge calculation, and fitting method (Clauset et al., 2009; Hayasaka and Laurienti, 2010; Zucca et al., 2019). For example, the earliest studies usually used the least-square fitting method on log–log plots to test whether a degree distribution is power law. This fitting method is systematically biased and does not take into account the goodness of fit and selection of prospective degree distributions (Clauset et al., 2009). Another study comparing functional brain networks at multiple resolutions found that although the degree distributions of all networks followed the power law with exponential cutoff, the higher the resolution (up to the voxel level), the more the distribution tended to be a power law (Hayasaka and Laurienti, 2010). In summary, in this study, we aimed to use a generally applicable, easily understood approach to discuss degree distribution of functional brain networks in rsfMRI. We used the Pearson association of FC to construct binary networks at the finest voxel level. To avoid the flaws of the least-squares, the maximum likelihood estimation and loglikelihood ratio methods were used to estimate and compare proposed models. Our results suggest that the short-tailed Weibull distribution is superior to the other two models in all generated networks.

The second major finding showed decreased Weibull β parameters of the global degree distribution in the whole-brain network and all seven functional subnetworks in aMCI. These nodal degrees were computed from themselves to all the other nodes in the whole brain. The calculation of the degree distribution was based on the strength of functional connectivity between paired voxels. Therefore, these findings are at least partially in agreement with previous reports that found alterations in resting-state connectivity in aMCI. Specifically, two meta-analyses reported increased connectivity in default mode, salience, and limbic networks, while decreased connectivity in default mode, frontoparietal, visual, and limbic networks (Li et al., 2015a; Badhwar et al., 2017). Another study that explored topological pattern changes of brain networks in aMCI reported decreased nodal centrality in the medial temporal lobe and increased nodal centrality in the occipital regions (Liu et al., 2012). Here we extended these findings to the network level, showing that the overall topology features of these functional subnetworks have changed in aMCI.

The underlying nature of the decrease in Weibull β parameter in aMCI is unclear. The Weibull distribution used here that describes the degree distribution of functional brain networks is a two-parameter model. Although no statistically significant between-group differences were found for the second γ parameter, it is difficult to determine the specific variation of the curve on the CCDF plot based on just one β parameter. The β parameter is the shape factor of the Weibull distribution. Its slight decrease may be thought of as a small shifting of the degree distribution from exponential to power law distribution, reflecting an increase in the number of hub nodes in the network. One possible explanation is that the decrease in Weibull β parameter reflects less efficient neural network activity. According to the dedifferentiation hypothesis, inefficient neural processing results in age-related brain functional changes that lead to more diffuse brain activity (Dennis and Cabeza, 2011). In line with the dedifferentiation hypothesis, a study found that age-related decreased modularity of resting-state FC within networks and increased inter-network connectivity in elderly cognitively healthy subjects (Geerligs et al., 2015). Another study showed increased number of FC connections in aMCI and AD compared to HC. The number of connections peaked in aMCI and is significantly higher compared to AD. Furthermore, increased strength of FC was found for connections that spanned different functional clusters were identified, including the FP network, the posterior DM network, the medial temporal lobe subsystem, and a subcortical cluster (Zhang et al., 2016). The human brain has the capacity to buffer or reserve itself against some extent of the changes brought on by aging and disease (Staff, 2012). It is possible that this more diffuse, less efficient neural processing may require an increase in the strength or the number of FC, with compensatory recruitment of additional neural resources to try to maintain task performance in early stage of AD (Grady et al., 2003; Dickerson et al., 2004). An alternative explanation is that the increase in FC results from increased deposition of Aβ (Elman et al., 2014; Huijbers et al., 2015). However, this hypothesis is still in doubt. The present study did not collect Aβ from the subjects, and therefore, no Aβ-related experimental manipulation was involved.

The third major findings found that the abnormal decrease in the value of Weibull β parameter was associated with reduced cognitive performance in aMCI. These findings are consistent with previous reports of increased DM network connectivity contributes to semantic memory deficits in MCI patients (Gardini et al., 2014). The abnormal increase in the strength of FC, not confined to the DM network but connected between the FP network and medial temporal lobe subsystem, was found to be associated with reduced episodic memory performance in MCI and AD (Zhang et al., 2016). Task-related studies have also shown that MCI patients had enhanced activation in the hippocampus (Dickerson et al., 2004) and its association with faster subsequent cognitive decline in MCI (Miller et al., 2008). Although most of the previous findings were related to the DM network, complex cognitive functions, such as memory, are distributed across several resting-state networks (Li et al., 2015b). Here we addressed the associations between changes in nodal degree distributions and cognitive ability at the functional network level. Negative correlations were found between the cognitive ability and the Weibull β parameters of several subnetworks' global degree distributions in aMCI. Notably, we focus on the number of FC-based connections for each node in the network, with each connection potentially linking to other functional networks. In other words, increase in the number of hub nodes in these networks is associated with cognitive decline in aMCI. Thus, the inverse association may reflect a failed compensatory attempt to recruit additional neural resources to maintain task performance. These results suggest that decrease in the Weibull β parameter characterizing the functional brain network is detrimental to cognitive performance in aMCI.

It is important to acknowledge the potential limitations of our study. Firstly, the number of subjects used in this study was relatively small, which may lead to potential statistical instability. Secondly, the determination of the connections during network construction will have an influence on the results. We used absolute thresholds so that connections that surpass the fixed connectivity strength were kept and set to 1. However, there is no consistent standard for the selection of threshold. In this study, the selection was based on the small-world characteristic and the integrity of the network. The networks generated with multiple thresholds were all well-fitted by the Weibull distribution. For validity reasons, we performed a FCS analysis, using the weighted degree centrality to avoid the selection of thresholds. The results also revealed enhanced degree centrality of several hub regions in aMCI (Supplementary Figure 3), which is consistent with the results of reduced Weibull β parameters using the absolute thresholds. Additionally, in rsfMRI, the spontaneous brain activation is sampled without reference to external tasks, so its interpretation is inherently less well-understood. Therefore, we attempted to frame the study in a hypothesis-driven manner, focusing on functional subnetworks derived from a data-driven approach based on 1,000 participants. However, the combination of resting-state and specific task-related fMRI studies would be important for future researches.

Overall, the current results on the altered degree distributions of functional brain subnetworks support that the degree distribution gives a window to evaluate the neural network topology underlying cognitive performance. This study offers a method for designing resting-state analysis to assess variations in degree distribution for providing insight into cognitive decline in aMCI. Degree distribution is currently not established as a biomarker for neuroimaging. Longitudinal studies are needed to examine the value of degree distribution to predict subsequent cognitive decline.

## Data Availability Statement

The raw data supporting the conclusions of this article will be made available by the authors, without undue reservation.

## Ethics Statement

The studies involving human participants were reviewed and approved by the Medical Research Ethics Committee of Peking University Sixth Hospital, Beijing, China. The patients/participants provided their written informed consent to participate in this study.

## Author Contributions

YZ designed the study, performed the statistical analysis, interpreted the results, and wrote the manuscript. XC, ZW, TX, and XW carried out the data collection. XL pre-processed the rsfMRI data. YS and WZ revised the manuscript. HW carried out the data collection, provided advices, and revised the manuscript. All authors contributed to the article and approved the submitted version.

## Conflict of Interest

The authors declare that the research was conducted in the absence of any commercial or financial relationships that could be construed as a potential conflict of interest.
