# Supplementary material for: Altered Weibull Degree Distribution in Resting-State Functional Brain Networks Is Associated With Cognitive Decline in Mild Cognitive Impairment
Source: Front Aging Neurosci. 2021 Jan 5;12:599112. doi: 10.3389/fnagi.2020.599112 (PMC7814317; doi:10.3389/fnagi.2020.599112)
Supplement: Supplementary file 1 [file Data_Sheet_1.docx]

Supplementary Material

# Supplementary Figures


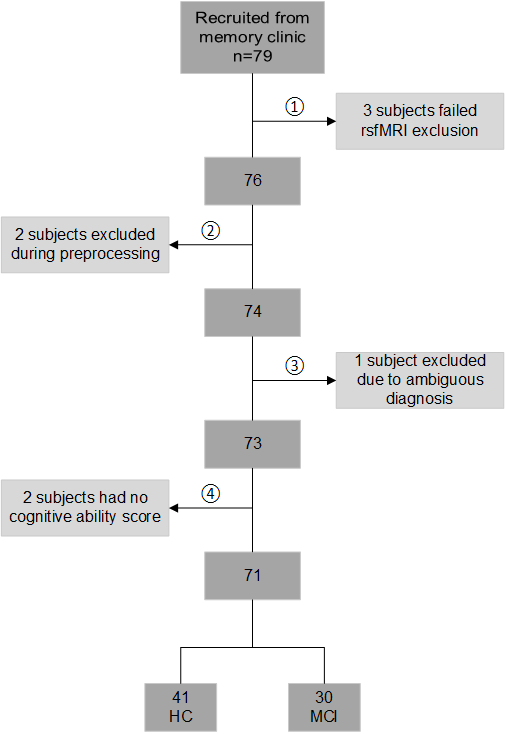


**Supplementary Figure 1.**

Flow chart of subject selection. Among the seventy-nine subjects with rsfMRI at baseline, eight subjects were excluded for the following reasons: (1) three subjects were excluded because of protocol violation or dropout, (2) two subjects had to be excluded, due to signal loss in the fMRI scan or motion artifact, (3) one subject was excluded due to ambiguous diagnosis information, (4) two subjects were excluded because they had no cognitive ability score.


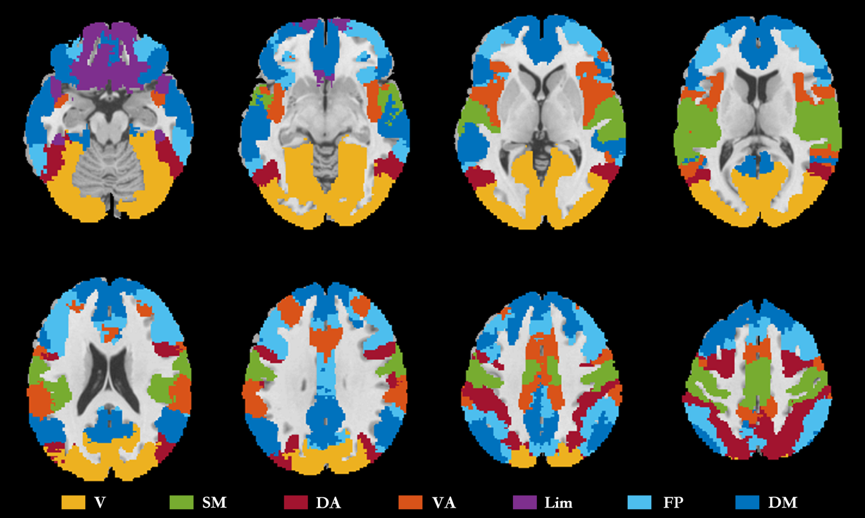


**Supplementary Figure 2.**

Seven functional subnetworks correspond to the meta-analytically determined brain areas based on the rsfMRI data from 1,000 participants and a data-driven clustering approach (Thomas Yeo et al., 2011). V, visual; SM, somatomotor; DA, dorsal attention; VA, ventral attention; Lim, limbic; FP, frontoparietal; DM, default model.

**
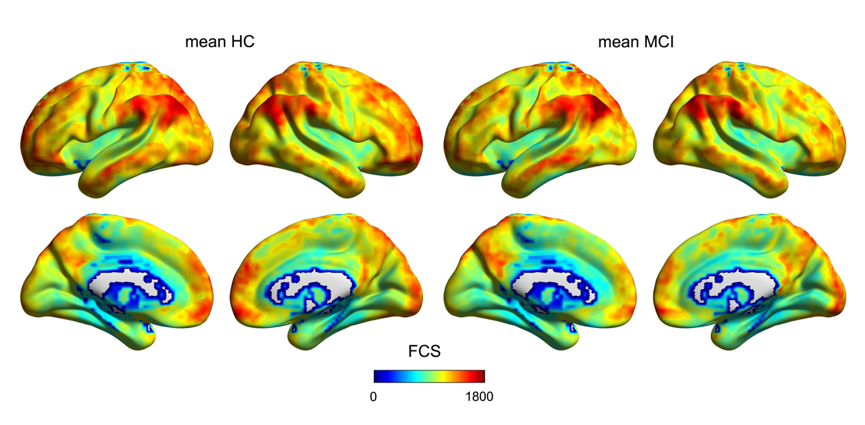
**

**Supplementary Figure 3.** Mean FCS maps in the HC and the aMCI groups, with a correlation threshold $r_{0}=0.2$ to eliminate weak correlation possibly arising from noise. The FCS values were mapped on the cortical surface by using BrainNet Viewer (Xia et al., 2013). FCS, functional connectivity strength; HC, cognitively healthy; aMCI, amnestic mild cognitive impairment.


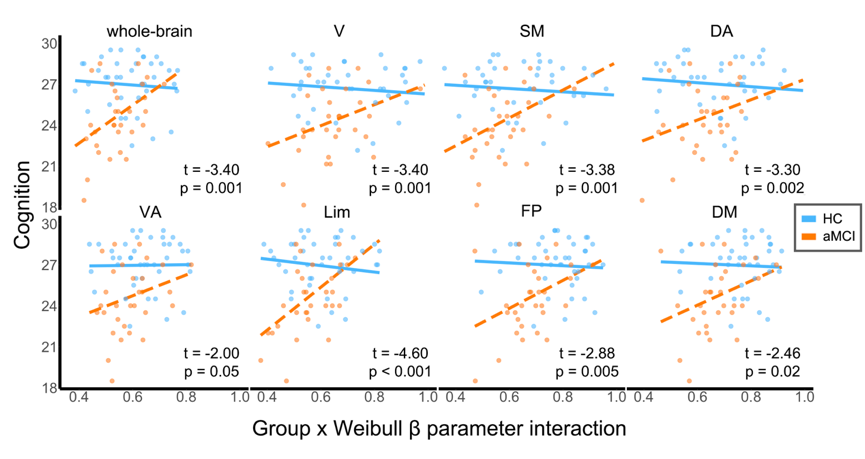


**Supplementary Figure 4.** Regression plots of the interactions between Diagnostic Group $\times$ Weibull β parameter for the association with cognitive ability scores, in the whole brain network and seven subnetworks (correlation threshold T = 0.4). V, visual; SM, somatomotor; DA, dorsal attention; VA, ventral attention; Lim, limbic; FP, frontoparietal; DM, default model; aMCI, amnestic mild cognitive impairment.

**Reference**

Thomas Yeo, B., Krienen, F.M., Sepulcre, J., Sabuncu, M.R., Lashkari, D., Hollinshead, M., Roffman, J.L., Smoller, J.W., Zöllei, L., and Polimeni, J.R. (2011). The organization of the human cerebral cortex estimated by intrinsic functional connectivity. *Journal of neurophysiology* 106, 1125-1165.

Xia, M., Wang, J., and He, Y. (2013). BrainNet Viewer: a network visualization tool for human brain connectomics. *PloS one* 8: e68910.
